# Balancing ethical norms and duties for the introduction of new medicines through conditional marketing authorization: a research agenda

**DOI:** 10.3389/fmed.2024.1408553

**Published:** 2024-06-24

**Authors:** Mariia V. Maksimova, Ghislaine J. M. W. van Thiel, Yke Tromp, Rosan Lechner, Johannes J. M. van Delden, Lourens T. Bloem

**Affiliations:** ^1^Department of Bioethics and Health Humanities, Julius Center for Health Sciences and Primary Care, University Medical Center Utrecht, Utrecht, Netherlands; ^2^Department of Clinical Genetics, Erasmus Medical Center, Rotterdam, Netherlands; ^3^Division of Pharmacoepidemiology and Clinical Pharmacology, Utrecht Institute for Pharmaceutical Sciences, Utrecht University, Utrecht, Netherlands

**Keywords:** conditional marketing authorization, research ethics, clinical ethics, expedited regulatory pathways, unmet medical need, uncertainty, informed consent, European Medicines Agency

## Abstract

The European Medicines Agency’s conditional marketing authorization (CMA) aims to expedite patient access to medicines for unmet medical needs by shifting a part of the drug development process post-authorization. We highlight ethical issues surrounding CMA, comprising (i) the complexity of defining unmet medical need; (ii) poor understanding of CMA and its impact on informed consent; (iii) hope versus unrealistic optimism; (iv) implications of prolonged post-authorization studies and potential patient harm; (v) rights and duties of patients surrounding participation in post-authorization studies; (vi) access to previously authorized CMA medicines; and (vii) the “benefit slippage” phenomenon, defined as the gradual shift of strict criteria to less strict criteria. We propose a comprehensive research agenda to address these ethical issues, and stress the need for multi-stakeholder engagement to ensure patient-centered use of CMA.

## 1 Introduction

**FIGURE 1:**
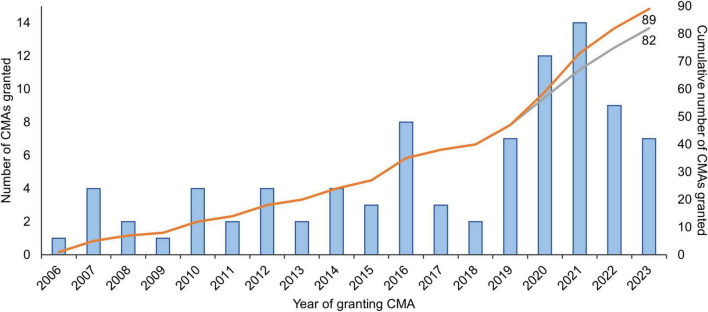
Number of medicines granted conditional marketing authorization in 2006-2023 in the European Union. The gray curve excludes the seven coronavirus disease 2019 (COVID-19) vaccines and treatments that were granted CMA (*N* = 2 in 2020; *N* = 4 in 2021; and *N* = 1 in 2022). Adapted from Bloem et al. (3) and updated with data from the European Commission’s Union Register of medicinal products (4). CMA, conditional marketing authorization.

To enable early access to promising medicines for patients with unmet medical needs–such as rare diseases or poorly treatable cancers–regulatory authorities have implemented expedited pathways that allow marketing authorization of medicines based on preliminary (“non-comprehensive”) data. Expedited pathways are in place in for example the European Union (EU), the United States (US), Japan, Canada, and other countries (1). In the EU, the European Medicines Agency’s (EMA) conditional marketing authorization (CMA) has been in place since 2006 (Box 1). Until the end of 2023, 89 CMAs were granted, of which almost half were granted in the last four years (*N* = 42, 47%; Figure 1).

**BOX 1 Characteristics of the conditional marketing authorization in the European Union (2).**

In the European Union, medicines can be granted conditional marketing authorization (CMA) based on non-comprehensive data when they are (i) intended for treatment, prevention or diagnosis of a seriously debilitating or life-threatening disease, (ii) designated an orphan medicine by the European Commission, or (iii) to be used in emergency situations, such as the coronavirus disease 2019 (COVID-19) pandemic. In addition, the following requirements should be met:

i. The benefit-risk balance of the medicine is considered positive based on the available evidence;
ii. It is likely that the developer can provide comprehensive data in a timely manner to resolve important uncertainties;
iii. An unmet medical need is expected to be fulfilled by the medicine; and
iv. The benefits to public health of the immediate availability of the medicine outweigh the risks inherent in the fact that additional data are still required.

To facilitate that comprehensive data become available and important remaining uncertainties about safety and efficacy are resolved, post-authorization studies called “specific obligations” are imposed as legally binding conditions of the CMA. Each year, the developer must submit a report describing the status of the specific obligations, after which the CMA can be renewed for another year. A continued positive benefit-risk balance is critical for maintaining the CMA. Upon completion of the specific obligations, the CMA can be converted to a standard marketing authorization.

The CMA is distinct from the authorization under exceptional circumstances for which comprehensive data are not expected to become available at all.

CMA aims to provide patients with early access to promising medicines through regulatory flexibility. It can offer a valuable opportunity for patients who often have no other options to meet their medical need. On the other hand, CMA comes with heightened uncertainty about benefits and risks and impacts several aspects of the drug development process. Moreover, in Europe, actual patient access is often dependent on national pricing and reimbursement processes following authorization. In this paper, we present an overview of ethical issues relevant to CMA and propose a research agenda to help further the responsible introduction of new medicines granted CMA in clinical practice and the use of CMA as a regulatory tool.

## 2 From CMA requirements to ethical issues

The CMA pathway forms a unique context in which urgent patient needs must be balanced with ensuring the safety and efficacy of medicines. This context builds on three ethically relevant characteristics of CMA (Box 1). The first characteristic is unmet medical need. The CMA’s primary aim is to provide patients with unmet medical needs with early access to promising medicines. Therefore, the main ethical driver for the CMA is the ethical principle of beneficence, which entails the duty to promote the health and wellbeing of patients with unmet medical needs. The second characteristic is uncertainty about the benefit-risk balance. The CMA introduces risks inherent to the reliance on initial, non-comprehensive data about the safety and efficacy of a new medicine. The third characteristic comprises mandatory post-authorization studies to obtain comprehensive data and resolve important remaining uncertainties (hereafter CMA studies). During these studies, patients in clinical practice face increased uncertainty about benefits and risks compared to patients treated with medicines granted standard marketing authorization. The initial assessment of the benefit-risk balance is not always confirmed with the emerging data from CMA studies (3, 5). As a result, medicines’ indications may be restricted or entire marketing authorizations withdrawn, revoked or not renewed. For example, the third-line treatment indication for ovarian cancer was recently removed for rucaparib (Rubraca) (6, 7), as well as the treatment indication for RET mutation-negative medullary thyroid carcinoma for vandetanib (Caprelsa) (8). The renewal of the marketing authorization of ataluren (Translarna) for Duchenne muscular dystrophy is currently (June 2024) under discussion (9), while the marketing authorization of belantamab mafodotin (Blenrep) for multiple myeloma was previously not renewed (10, 11) and that of olaratumab (Lartruvo) for soft tissue sarcoma revoked (12, 13).

The combination of non-comprehensive evidence at initial marketing authorization and the subsequent mandatory CMA studies moves a part of the drug development process, traditionally performed in the highly controlled research setting, into regular clinical practice. Medicines that not (yet) meet the scientific standard of comprehensive evidence are nonetheless released in the clinical setting in which different actors make decisions and the ethico-legal regulations for treatment apply alongside those for post-authorization research. This results in a hybrid situation in which the medicine is still in the research stage as well as part of (standard) clinical care. Current ethical norms and regulations guiding the drug development process are not optimally suited for such hybrids.

## 3 Ethical issues related to CMA

**FIGURE 2:**
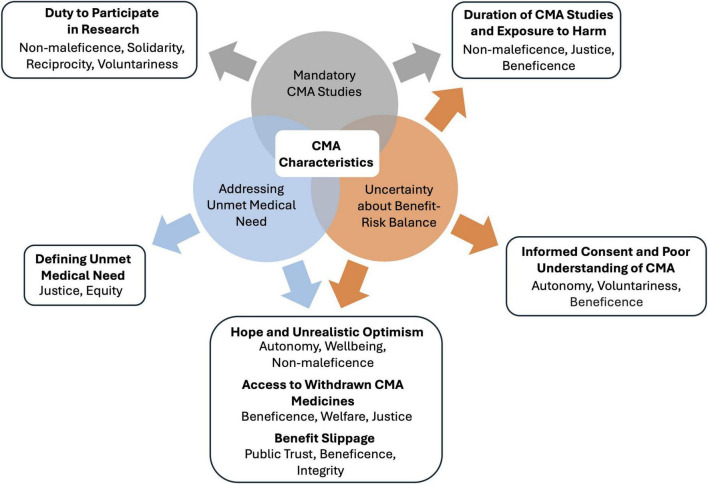
Relationships between characteristics of conditional marketing authorization (CMA) and ethical issues. CMA requirements result in three ethically relevant characteristics of CMA: addressing an unmet medical need, uncertainty about the benefit-risk balance, and mandatory CMA studies. These characteristics and their intersections provoke ethical issues, which are listed in bold. Below each ethical issue, the implicated ethical principles and values are shown. CMA, conditional marketing authorization.

Ethical issues related to CMA (Figure 2) are described below and derived from an in-depth analysis of the context of CMA and its implications for the ethical delivery of healthcare. The issues described in this paper are highlighted for their direct impact on CMA stakeholders and their potential to inform and refine ethical guidelines and policy development. Therefore, for each ethical issue, needs for future inquiry are discussed, establishing a comprehensive research agenda for CMA. Although the analysis focuses on ethical issues in the context of CMA, some may also apply to other contexts.

### 3.1 Defining unmet medical need

Defining “unmet medical need” for CMA presents an ethical challenge as this definition impacts priority setting in diseases and patient groups for expedited regulatory pathways. The current EU regulatory framework identifies unmet medical need as “a condition for which there exists no satisfactory method of diagnosis, prevention or treatment authorized in the Union or, even if such a method exists, in relation to which the medicinal product concerned will be of major therapeutic advantage to those affected” (2). Yet, interpretations vary widely among stakeholders and countries. Vreman et al. (14) identified 16 distinct definitions. Of these, six definitions considered the severity or burden of the disease, and one considered the size of the patient population affected (14).

Various stakeholders have suggested changes to the definition of unmet medical need (15–17). In the light of the reform of the EU pharmaceutical legislation, a definition based on meaningful or substantial reduction in morbidity and mortality was recently proposed (18, 19). However, such proposals may introduce even more ambiguity because of the subjective nature of what is considered a “substantial reduction.” The current debate shows that the assessment of unmet medical need is highly dependent on the scope and the value framework of each stakeholder (14, 17).

Given the significant influence of the definition of unmet medical need on healthcare priorities and resource allocation, researching how to refine this definition is important. Such research should aim to include perspectives of different stakeholders. One way to achieve this could be through multi-criteria decision analyses (20). Additionally, comparative studies across different healthcare systems could provide insights into how varying definitions of unmet medical need impact healthcare delivery and innovation.

### 3.2 Informed consent and poor understanding of CMA

Voluntary informed consent is a fundamental ethical requirement for clinical care as well as medical research. To fulfill this requirement, patients, physicians, and researchers need to understand the uncertainties surrounding the benefit-risk balance of medicines granted CMA. Studies show that patients and healthcare professionals often misunderstand the disparities in data that may underlie new medicines. For example, Schumacher et al. (21) found that in the context of experimental cancer treatment trials, around 80% of patients did not understand that the benefits of treatment were uncertain and that participation was associated with additional risks. Similarly, Woloshin and Schwartz (22) noted widespread misconceptions among the public about approvals by the US Food and Drug Administration (FDA), with many believing that such approvals guarantee efficacy or the absence of severe side effects.

Physicians also need to have sufficient understanding to communicate effectively with their patients (23, 24). Research shows, however, that physicians often misinterpret the robustness of evidence used for regulatory decision-making by the FDA, leading to overestimated benefits and underestimated risks (23, 25–27). Moreover, uncertainties about the benefit-risk balance are seldom communicated by regulators to physicians, patients, or the public (28).

This jeopardizes the physician’s duty to adequately explain information and subsequently impacts voluntariness and the quality of the patient’s informed consent (29). Research should investigate the understanding of CMA among European patients and physicians, including the broader perceptions of EMA authorizations. In addition, it is important to further study and implement existing strategies to improve the informed consent process (30). Furthermore, there has been a call for attention to specific groups, particularly children, as specific guidelines in this area are currently lacking (31).

### 3.3 Hope and unrealistic optimism

A combination of dire need and the glimpse of a solution may foster unrealistic expectations of medicines granted CMA among patients and families. Scholars have debated the role of hope, optimism, and realism in access to investigational drugs (32, 33).

False or unrealistic optimism is a cognitive bias where individuals believe they are less likely to experience adverse outcomes and more likely to experience positive outcomes. Unrealistic optimism is distinct from being misinformed (as discussed in relation to informed consent) since it operates as an internal bias undermining the accurate appreciation of risks and benefits of a treatment. This can adversely affect patients’ health and wellbeing (34–36). In ethical terms, unrealistic optimism may compromise autonomy in decision-making which is characterized by intentionality and freedom from controlling influences. For instance, unrealistic optimism shapes how patients decide not only about treatment but also about other life plans, leading them to make choices they would not have considered if they had more realistic expectations (37).

On the other hand, some scholars suggest that unrealistic optimism is not as harmful as is often thought. They argue that unless such optimism leads to choices that are clearly misaligned with patients’ values and goals, it may not be detrimental to autonomy (38–41). Unrealistic optimism, experienced as hope, can have positive effects in healthcare, such as resilience and helping to endure adversity. Hope as resilience was a positive factor associated with recovery, and empowerment (42).

The distinction between beneficial and detrimental expectations is crucial, and conflating them overlooks the possible harms of false hope or optimism (43). For medicines granted CMA, this distinction is essential given their provisional benefit-risk balance, which may foster unrealistic patient expectations. To address this issue, further empirical research could serve as a starting point to gain more insight in relevant factors. As expectations, understanding, and hope are interlinked in medical decision-making, this research should be conducted alongside research on enhancing informed consent processes, as discussed in the previous section.

### 3.4 Duration of CMA studies and exposure to harm

The CMA of ataluren is currently under discussion after 10 years of CMA status and the indication of vandetanib was restricted after 11 years (8, 9). These cases highlight ethical concerns about the potential harms of prolonged use under CMA. These harms go beyond direct effects of the medicines (44). They also include the missed opportunity for alternative treatments, and the societal harm of inefficient resource allocation. In ethical terms, the timelines until the potential non-renewal and restriction of the CMAs for ataluren and vandetanib raise the question: Was the duty to minimize risk and avoid harm safeguarded sufficiently?

In contrast, the CMAs for olaratumab and belantamab mafodotin were revoked and not renewed after less than three and four years on the market, respectively (10–13). This variance highlights the challenges in determining the feasibility and acceptable duration of CMA studies. However, addressing these problems in the conduct of CMA studies requires a tailored approach. For instance, in rare diseases, patient recruitment challenges can cause delays. Adding pressing time frames could result in effective treatments being restricted, withdrawn, revoked or not renewed prematurely or put undue pressure on patients. It could also increase the uncertainty, when, for example, reliance on surrogate endpoints in clinical research would increase to shorten the time to study completion.

Further research should identify the ethical and social factors that define the optimal duration of CMA studies. This exploration should aim to inform a discussion on acceptable timeframes for the completion of CMA studies and the subsequent decision-making process regarding the conversion or withdrawal, revocation or non-renewal of CMAs, thereby prioritizing the perspectives of patients, physicians, and society.

### 3.5 Duty to participate in research

The need for efficient CMA studies to resolve uncertainties about safety and efficacy raises another ethical question: Should access to a medicine that is granted CMA come with a duty to participate in CMA studies? Advocates for such a duty argue that individuals who enjoy the benefits of the healthcare system have a moral responsibility to contribute to the medical knowledge that supports it (45–47). They posit that participation in biomedical research is a form of reciprocity rooted in justice and fairness, especially when it can prevent harm to others. Specifically, for medicines granted CMA, participation in research by patients who receive these medicines can accelerate the collection of comprehensive data, limit the use of ineffective or unsafe medicines and thereby safeguard the interests of other patients and optimize public resource allocation.

However, this stance encounters several ethical challenges. First, it may interfere with the principle of voluntariness of research participation—a cornerstone of research ethics underscored in international guidelines (48, 49). The question of voluntariness becomes particularly complex among vulnerable groups who may feel compelled to participate due to unmet medical needs. Framing this duty as contributing to the common good and as inherently beneficial could inadvertently boost hope and potentially unrealistic expectations. Second, CMA studies may involve additional medical procedures, which could be invasive or risky. It is unclear which level of risk or burden would be acceptable under a duty to participate.

Investigating how to shape a duty to participate in a way that avoids these ethical pitfalls could be the focus of further research. For instance, conceptualizing this duty as an act of solidarity in advancing medical practice rather than a forced obligation could shift perspectives. Solidarity, as suggested by Hollestelle et al. (50), is fostered not through imposition but by empowering individuals. This raises a compelling question for future research: How can we encourage patient participation in post-authorization studies in general and CMA studies in particular in an empowering way?

### 3.6 Access to previously authorized CMA medicines

The experience with restricting indications and withdrawing, revoking or not renewing CMAs due to unmet specific obligations is limited. Meanwhile, losing access to a medicine poses an under-discussed ethical issue in the conceivable scenario when individual patients demonstrate benefit despite a lack of evidence of a favorable benefit-risk balance at the population level.

This scenario shares similarities with post-trial access, as delineated in the Declaration of Helsinki’s Article 34 (49). The principles underpinning post-trial access—emphasizing individual welfare—offer a precedent for interpreting the right to access treatments proven beneficial on a personal level (51). One can suggest that, by analogy, there is *prima facie* right to individual arrangements after withdrawn, revoked or not renewed CMAs in case of proven personal benefit. However, this interpretation must be approached with caution, recognizing that individual patient improvements might be overestimated or caused by additional factors (52–54). Notably, regulating access to previously authorized CMA medicines may be difficult if guidelines continue to recommend this use while the medicine remains authorized for another (part of the) indication (55). Given the above, there is a need for research on ethically justified and reasonable arrangements for previously authorized CMA medicines, such as a tailored fade-out process.

### 3.7 Benefit slippage

The aim of CMA to help patients with unmet medical needs may result in so-called “benefit slippage.” Juth (56) identifies benefit slippage as the gradual shift of strict criteria for medical interventions to less strict criteria. He describes this phenomenon observed in neonatal screening programs, where the bar for including conditions lowers over time. This trend occurs because new conditions are added to the screening based on their similarity to previously included ones rather than direct health benefits (56). A similar phenomenon could arise in the context of CMA, where the drive to address unmet medical needs may lead to the acceptance of medicines with a higher level of uncertainty regarding their benefit-risk balance. This may erode original stringent criteria for authorization and undermine the primary aim of the regulatory framework: protecting public health. A previous study observed that applications for CMA in oncology have changed from the use of CMA as a “rescue option” to its proactive use by pharmaceutical companies, suggesting that these companies have learned what types of data are considered acceptable for CMA (3). The increasing use of single-arm trials and surrogate endpoints to support CMA has been a cause of concern, and their use is not limited to CMA (5).

Concerns about benefit slippage have also been expressed by, for example, Swedish governmental organizations. They fear that the European Commission’s proposals for increased use of expedited regulatory pathways will lower evidence standards (57). Future research should examine the phenomenon of benefit slippage in the context of CMA, including whether and how evidence standards may shift over time and the implications for patient care and policy-making.

## 4 Discussion

Our study of the ethical issues related to CMA reveals the merging of research and clinical practices as a root cause of ethical tension. Historically, these two domains have been distinct (54), but within the realm of CMA, they increasingly overlap, as CMA moves a part of the drug development process into clinical care. CMA is not alone in this trend; several other hybrid practices have emerged, such as highly individualized therapies or *N*-of-1 clinical strategies, highlighting a broader shift toward integrating research and clinical care (54, 58, 59). This overlap indicates the need for a joint ethical framework to navigate the complexities in such a hybrid healthcare setting, including ethical norms, guidelines, and regulations.

To further improve the regulatory and clinical practices of CMA, it is crucial to actively involve patients, clinicians, and ethicists. Their collaboration is essential in areas like defining unmet medical need, ensuring informed consent, and managing patient expectations. Empirical research can provide insights relevant to specific ethical complexities in the context of CMA. Optimal use of existing best practices (i.e., for informed consent) can also help diminish ethical tension. Addressing the complexities in optimizing CMA study durations, potential restriction, withdrawal, revocation or non-renewal of CMA, and the phenomenon of benefit slippage requires a collaborative ecosystem that comprises all stakeholders, including pharmaceutical companies and policymakers.

In conclusion, the research agenda proposed in this paper provides the starting point for fostering an ethically grounded and patient-centered approach to using CMA, prioritizing welfare and minimizing the risk of harm in the introduction and use of promising new medicines.

## Author contributions

MM: Conceptualization, Formal analysis, Investigation, Visualization, Writing – original draft, Writing – review & editing. GT: Conceptualization, Formal analysis, Funding acquisition, Investigation, Project administration, Supervision, Visualization, Writing – original draft, Writing – review & editing. YT: Formal analysis, Investigation, Writing – original draft, Writing – review & editing. RL: Formal analysis, Writing – review & editing. JD: Supervision, Writing – review & editing. LB: Conceptualization, Formal analysis, Investigation, Project administration, Supervision, Visualization, Writing – original draft, Writing – review & editing.
